# The Effect of Beta-Aminopropionitrile and Prednisolone on the Prevention of Fibrosis in Alkali Esophageal Burns: An Experimental Study

**DOI:** 10.1155/2013/574260

**Published:** 2013-12-11

**Authors:** Kurtulus Aciksari, Hakan Teoman Yanar, Gulcin Hepgul, Dogac Niyazi Ozucelik, Fatih Yanar, Orhan Agcaoglu, Mediha Eser, Gamze Tanriverdi, Hakan Topacoglu, Baris Murat Ayvaci, Halil Dogan, Kayihan Gunay, Cemalettin Ertekin, Ferudun Celikmen

**Affiliations:** ^1^Department of Emergency Medicine, Faculty of Medicine, Istanbul Bilim University, 34394 Istanbul, Turkey; ^2^Department of General Surgery, Istanbul Faculty of Medicine, Istanbul University, 34093 Istanbul, Turkey; ^3^Department of General Surgery, MoH Bagcilar Research and Training Hospital, 34200 Istanbul, Turkey; ^4^Department of Emergency Medicine, MoH Bakirkoy Dr. Sadi Konuk Research and Training Hospital, 34147 Istanbul, Turkey; ^5^Department of Histology and Embryology, Cerrahpasa Faculty of Medicine, Istanbul University, 34098 Istanbul, Turkey; ^6^Department of Emergency Medicine, MoH Istanbul Research and Training Hospital, 34098 Istanbul, Turkey; ^7^Department of Emergency Medicine, MoH Okmeydani Research and Training Hospital, 34384 Istanbul, Turkey; ^8^Department of Emergency, MoH Avcilar State Hospital, 34320 Istanbul, Turkey; ^9^Department of Emergency Medicine, MoH Kartal Lutfi Kırdar Research and Training Hospital, 34880 Istanbul, Turkey

## Abstract

*Objective*. The aim of this study was to investigate the efficacy of beta-aminopropionitrile (BAPN) and prednisolone on the prevention of esophageal damage and stricture formation after caustic esophageal burn. *Method*. Twenty-eight rats were divided into four equal groups. In groups 1, 2, and 3, caustic esophageal burns were generated by applying NaOH to the 1.5 cm segment of the abdominal esophagus. Group 4 was for the sham. Normal saline to group 1, BAPN to group 2, and prednisolone to group 3 were administered intraperitoneally as a single daily dose. *Results*. Treatment with BAPN decreased the stenosis index (SI) and histopathologic damage score (HDS) seen in caustic esophageal burn rats. The SI in group 4 was significantly lower compared with groups 1, 2, and 3. Group 2 had the minimum SI value in corrosive burn groups. The differences related to SI between groups 1, 2, and 3 were not statistically significant. The HDS was significantly lower in group 4 compared with groups 1, 2, and 3. The HDS in group 2 was significantly lower compared with groups 1 and 3. *Conclusion*. This study demonstrated that BAPN was able to decrease the development of stenosis and tissue damage better than prednisolone.

## 1. Introduction 

Exposure to caustic ingestions continues to be a serious problem in underdeveloped countries [1–3]. The incidence of corrosive agent ingestion ranges from 1/5000 to 1/26000 in the United States [4, 5]. Seventy percent of caustic injuries are located in esophagus [6]. Damage to the esophagus and stomach by caustic agents depends on the type, concentration, and form of those caustic agents and the duration of exposure [4, 5]. Following ingestion, solid or granular caustics often injure the oropharynx and proximal esophagus while liquid alkali ingestions are characterized by more extensive esophageal and gastric injuries. Severe intentional alkali ingestion may cause deep penetration into surrounding tissues with resultant multisystem organ injuries, including ocular, esophageal, and gastric perforation and necrosis of abdominal and mediastinal structures [7]. Animal data have shown decreased stricture formation with drugs that affect collagen deposition, including interferon-alfa-2b, octreotide, beta-aminopropionitrile (BAPN), N-acetylcysteine, and D-penicillamine [7, 8].

Few studies have demonstrated lathyrogens affecting the collagen fibril diameters and reducing intra-abdominal adhesions, anastomosis, and strictures [9–11]. BAPN is a compound known to cause lathyrism, a neurological disease by inhibiting lysyl oxidase, the enzyme that initiates cross-linkage formation in elastin and collagen; this action prevents the formation of cross-bonds, strictures, and collagen synthesis [12]. BAPN studies regarding the control of esophageal stenosis have been applied in 1972 by William et al. They were performed to test the hypothesis that esophageal stenosis can be prevented by altering cross-linking of newly synthesized scar collagen with the addition of mechanical bougienage, BAPN, and prednisolone treatment in the canine model [13, 14].

### 1.1. Goals of This Investigation

The aim of this experimental study was to investigate the effects of BAPN and prednisolone on the histopathological tissue damage and stenosis that develops after the esophagus is exposed to caustic substance.

## 2. Material and Methods 

### 2.1. Study Design and Settings

This experimental study was conducted at the Experimental Medicine and Research Center of Istanbul University Cerrahpasa Faculty of Medicine after approval from the Local Ethical Committee of the Turkish Republic Istanbul University for Animal Studies (Issue: 23, Decision Number: 23, 25/02/2010).

### 2.2. Experimental Animals

This study was conducted using twenty-eight Wistar albino rats weighting 200 to 250 grams. Before the study, all animals were kept in metal cages at 22°C with a 12-hour light/dark cycle for 10 days. The rats were starved for one night prior to laparotomy. After laparotomy, the animals were given free access to pellet feed and water and were maintained under laboratory conditions for twenty-eight days.

### 2.3. Chemical Esophageal Burn Model

Laparotomy was performed after the administration of intraperitoneal anesthesia with 40 mg/kg of thiopental sodium. In this study, a 1.5 cm segment of the abdominal esophagus was used; a 48 cm 6-F catheter was passed through the mouth and inserted into the upper segment of the abdominal esophagus. To prevent the leakage of corrosive agents into the stomach, the cardioesophageal junction was tied from the outside with 2/0 silk. To prevent the aspiration of the corrosive agent into the respiratory system, the esophagus was tied from the bottom of the proximal diaphragm with 2/0 silk. One mL of a 37.5% NaOH (pH = 12) solution was infused for 90 seconds and then aspirated. Subsequently, the burnt segment was irrigated with distilled water for 60 seconds. The proximal 2/0-suture was cut and drawn into the catheter with negative pressure. Subsequently, the distal 2/0-suture was cut, and the laparotomy was closed [15].

### 2.4. Experimental Groups

*Group 1 (Control)*. A 1 mL dose of 0.9% saline was administered intraperitoneally as a single daily dose to seven rats with corrosive esophagitis without any treatment for twenty-eight days.

*Group 2 (BAPN)*. After the initiation of corrosive esophagitis, a 500 mg/kg dose of BAPN was administered intraperitoneally to seven rats for twenty-eight days as a single daily dose. The time interval between induction of caustic injury and the intraperitoneal instillation of BAPN was ten minutes.

*Group 3 (Prednisolone)*. After the initiation of corrosive esophagitis, a 1 mg/kg dose of prednisolone was administered intraperitoneally to seven rats for twenty-eight days as a single daily dose. The time interval between induction of caustic injury and the intraperitoneal installation of BAPN was ten minutes.

*Group 4 (Sham)*. No corrosive esophageal burn was initiated after laparotomy. A 1 mL dose of 0.9% saline was administered intraperitoneally to seven rats as a single daily dose for twenty-eight days.

In our treatment schedule decision we have considered the ambulance transport times in Turkey. The median ambulance transport time in Turkey was approximately 10 minutes [16].

### 2.5. Collection of Samples

At the end of the experimental period, the rats were sacrificed by decapitation under anesthesia and two cm of esophageal tissue was excised for histological study. The proximal portion of the damaged segment was placed in 10% neutral formaldehyde for histological studies.

### 2.6. Histological Analysis

In our study, we choose the histopathological tissue damage score (HDS) and stenosis index (SI) as a method of evaluation. SI is used to determine the degree of stenosis in hollow organs and to determine the severity of esophageal stenosis. HDS is used to determine the severity of injury and collagen deposition in esophagus tissue.

The esophageal tissue samples were fixed in 10% neutral formaldehyde and stored at 4°C. Fixed tissue samples were applied routinely by paraffin embedding technique. The esophageal sections of 5 *μ*m in thickness were taken and stained with van Gieson and Azan [17, 18]. Preparations were evaluated by a bright field microscope and were photographed (Olympus BX61, Tokyo, Japan). All the samples were stained with Azan and magnifications are ×10 for Figures 3(a), 3(c), 3(e), and 3(g) and ×40 for Figures 3(b), 3(d), 3(f), and 3(h).

### 2.7. Evaluation of the HDS

Preparations were examined for HDS. Tissues were scored on a scale (none, 0; mild, 1+; marked, 2+) in three categories (collagen deposition in the submucosa, damage to the muscularis mucosa, and damage and collagen deposition in the tunica muscularis) for a total score of 0–5 [19].

### 2.8. Evaluation of the SI

A light microscope in ocular micrometer (Olympus BX61, Tokyo, Japan) magnification with the scanning objective (×40) was used to visualize the samples and to measure the mean esophageal wall thickness and lumen diameters from four different locations by two histologists. For SI calculations, the esophageal wall thicknesses were measured from two different areas, and the mean was calculated as [*A*_*o*_ = (*A*_1_ + *A*_2_)/2]. The lumen width was measured with two different linear lines, and the mean value was computed using the following equation: [*B*_*o*_ = (*B*_1_ + *B*_2_)/2]. SI is calculated as [Wall Thickness Mean (*A*_*o*_)/Lumen Diameter Mean (*B*_*o*_) = SI]; therefore, in this study, the following formula was used: (SI) = [Wall Thickness (*A*_1_ + *A*_2_)/2]/[Lumen Diameter (*B*_1_ + *B*_2_)/2] [20, 21]. The investigator who performed these measurements was unaware of the experiment.

### 2.9. Statistical Methods

All statistical analysis was performed using SPSS statistical software (release 21.0, SPSS, Chicago, IL, USA). The data were expressed as mean ± standard deviation (S.D.), minimum-maximum and median. The distribution of variables was checked with Kolmogorov-Smirnov test. To determine significant differences between the groups, a one-way ANOVA was performed followed by Tamhane T2 tests. Results were considered statistically significant at *P* < 0.05.

## 3. Results

**Figure 1:**
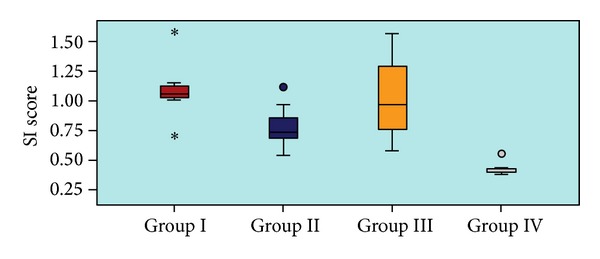
The histological appearances and parameters for calculating the SI.

The histological appearances and parameters for calculating the SI are shown in Table 1 and Figure 1.

There was a significant difference between the groups (Control, BAPN, Prednisolone, and Sham) (*P* < 0.05).

The SI in group 4 (0.44 ± 0.06) was significantly lower compared with group 1 (1.09 ± 0.26), group 2 (0.79 ± 0.19), and group 3 (1.03 ± 0.38)(*P* < 0.05).

Group 2 (BAPN) had the minimum SI value compared with the other corrosive burn groups (Control and Prednisolone). Nevertheless, differences related to SI between Groups 1 (Control), 2 (BAPN), and 3 (Prednisolone) were not statistically significant (*P* > 0.05).

**Figure 2:**
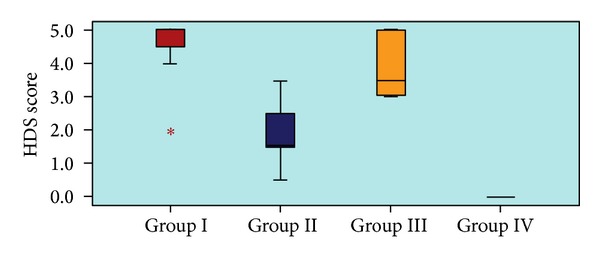
The histological appearances and parameters for calculating the HDS.

The histological appearances and parameters for calculating the HDS are shown in Table 1 and Figure 2.

There was a significant difference between the groups (Control, BAPN, Prednisolone, and Sham) in terms of the HDS (*P* < 0.05).

The HDS was significantly lower in group 4 (0.00 ± 0.00) compared with group 1 (4.43 ± 1.13), group 2 (1.93 ± 1.02), and group 3 (3.93 ± 1.02), (*P* < 0.05).

The HDS in group 2 (BAPN) was significantly lower compared with groups 1 (Control) and 3 (Prednisolone) (*P* < 0.05).

Group 2 (BAPN) had the minimum HDS value compared with the other corrosive burn groups (Control and Prednisolone). Although, there was a significant difference in terms of the HDS between Group 1 and Group 2 (*P* < 0.05), the mean HDS difference between Group 1 and Group 3 was not found statistically significant (*P* > 0.05).

**Figure 3:**
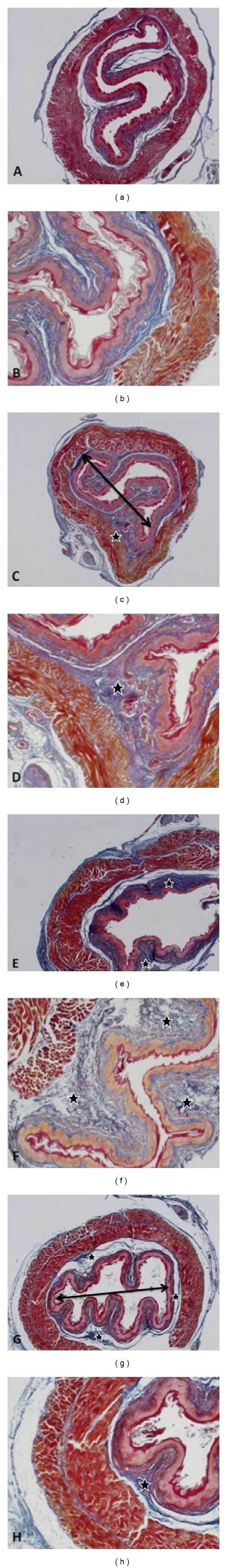
In the sham group, the normal histologic structure is visualized ((a)-(b)). The corrosive burn group demonstrates a constricted lumen (double arrow) and increased submucosal connective tissue (★), ((c)-(d)). And also, the prednisolone-treated group demonstrates persistent histological changes with increased submucosal connective tissue ((e)-(f)) like the corrosive burn group. The BAPN-treated group demonstrates histological findings that are similar to the sham group ((g)-(h)). All the samples were stained with Azan and magnifications are ×10 for (a), (c), (e), and (g) and ×40 for (b), (d), (f), and (h).

Normal histological structure was observed in the sham group (Figures 3(a) and 3(b)). The histological samples of the corrosive burn group demonstrated a constricted lumen and increased submucosal connective tissue (Figures 3(c) and 3(d)). In the prednisolone-treated group, histological changes were persistent and the increased submucosal connective tissue was still present (Figures 3(e) and 3(f)). The histological samples of the BAPN-treated group demonstrated similar characteristics to the sham group (Figures 1(g) and 1(h)).

The results indicate that BAPN treatment significantly reduced the HDS while prednisolone treatment did not. The HDS for fibrosis development was significantly lower in BAPN-treated rats than in prednisolone-treated rats.

Also, the results demonstrated that BAPN treatment reduced the SI better than prednisolone treatment in corrosive burn groups although did not significantly.

In this study, all animals survived the entire study, and there was no complication in the sham-operated group.

## 4. Discussion

Many household and industrial chemicals have caustic potential. Following ingestion, solid or granular caustics often injure the oropharynx and proximal esophagus. The ingestion of caustic substances causes immediate necrosis, commonly leading to transmural inflammation and results in corrosive esophagitis [7]. Caustic substances cause both functional and histological damage on contact with body surfaces. SI and HDS were used in previous studies to demonstrate the esophageal strictures [22–24].

To prevent stricture formation, many experimental studies have been performed to evaluate the therapeutic efficacy of agents such as sucralfate, trimetazidine, pentoxifylline, 3-amino-benzamide (3-AB), and ketotifen [22–25].

It is known that an increase in the inflammatory reaction in the connective tissue as a result of caustic burns results in increased fibroblast production, collagen production, and scar formation [26, 27].

In a randomized retrospective study, it has been shown that the prevalence of stricture development in patients with advanced stage corrosive esophagitis could be reduced with sucralfate treatment [25]. It has also been demonstrated that related to SI the combination of trimetazidine and pentoxifylline is more beneficial than pentoxifylline alone and is more efficacious in preventing stricture formation after corrosive burns [22]. 3-AB has also been demonstrated to have preventive effects on fibrosis formation and antioxidant enzyme activity in rats with corrosive esophagitis. The SI in the untreated group was significantly higher than in the sham-operated and 3-AB treatment groups [23]. In addition, ketotifen has been demonstrated to have preventive effects on fibrosis in rats with corrosive esophagitis. The SI in the untreated group was significantly increased compared with the sham laparotomy and the ketotifen-treated groups [24].

The literature is unclear regarding the use of corticosteroid therapy to prevent the development of esophageal stricture after the ingestion of corrosive substances. While, in some studies, it has been reported that stricture development is between 0% and 25% after corticosteroid treatment of esophageal burns versus 88% for cases in which corticosteroids are not used, other studies indicate that corticosteroids are not beneficial [28–32].

BAPN leads to lathyrism by inhibiting lysyl oxidase, which is an important enzyme in the synthesis of collagen and elastin. Mature collagen is not affected. Experimental animal studies have found that the most effective mechanism of controlling scar formation is with controlled lathyrism induced by BAPN treatment. Highly purified BAPN appears generally nontoxic and is capable of exerting a highly selective and significant lathyrogenic effect on the healing wound [33].

In a study conducted on scar formation in colon anastomoses and the effects of iatrogenic medications, it was demonstrated that the local and systemic use of BAPN reduces postoperative intra-abdominal adhesions and significantly reduces the thickness of granulation tissue [34]. Doolin et al. detected regression of stenosis when a 40 mg/day dose of BAPN was used to treat dogs with subglottic stenosis that developed after prolonged intubation [35]. In a study in which wound contraction was treated with local injections of BAPN and D-penicillamine, Joseph et al. showed that collagen accumulation in the rat skin was reduced; this study demonstrated that local administration of BAPN in combination with D-penicillamine reduces wound contraction and prevents contracture development [36].

In 1972, Davis et al. have demonstrated that inhibiting lysyl oxidase activity prevents stenosis in dogs with acute caustic esophageal lye burns [13]. BAPN alone, without addition of mechanical bougienage, is as effective in preventing esophageal stenosis as large doses of systemic steroid hormones and biweekly bougienage.

A year after this study, Madden et al. created an experimental esophageal lye burn with installation of 20% sodium hydroxide. Treating with bougienage and prednisolone improved the ability of dogs to swallow but did not produce a significant increase in esophageal diameter nor restore normal eating patterns. In contrast, treating with bougienage and BAPN produced a significant increase in esophageal diameter and restored normal deglutition [14].

The SI and HDS are important indicators of esophagus stenosis in caustic esophageal burns. In our study, comparison of the corrosive burn groups, the SI and HDS in untreated group was significantly increased compared with BAPN-treated and prednisolone-treated groups.

In this study, we found that prednisolone treatment did not significantly decrease the SI and HDS compared with the untreated group. The prednisolone treatment was not enough to reduce the stenosis and tissue damage in corrosive esophagitis development of esophageal strictures like in other studies [32, 33].

In our study, it has been demonstrated that BAPN reduced HDS and SI better than prednisolone in rats with corrosive esophageal burns. These findings suggest that BAPN can have a protective effect on the decrease of stenosis by reducing tissue damage. Similarly, Madden et al. have found that administration of BAPN produced a significant increase in esophageal diameter and restored normal deglutition. Once stenosis had been corrected, strictures did not recur in BAPN treated dogs.

In our rat model, the differences related to SI between control, BAPN-treated, and Prednisolone-treated groups were not statistically significant. Clinically, we observed that rats in the three groups behaved swallowing similarly throughout the study period.

In our experimental study, there was no significant difference between BAPN-treated and prednisolone-treated groups in terms of SI, compared with control group. SI is used for the indicator of esophageal stenosis and strictures. The results also indicate that BAPN treatment significantly reduced the HDS while prednisolone treatment did not. HDS assessment could be more valuable than SI in deep, muscle penetrating burns of esophagus.

In our study, HDS in terms of the role of prevention of the fibrosis development of the group being treated by administrating BAPN was lower compared to the group in which prednisolone was administrated.

It has been shown that the healing effect of BAPN on corrosive esophageal burns in terms of histological assessment was meaningful in this study.

It has been demonstrated that BAPN reduced HDS and SI in rats with corrosive esophageal burns. This experimental study suggests that BAPN can have a protective effect on the development of fibrosis by reducing tissue damage.

## 5. Conclusion

BAPN was found to be more effective in regression of the esophageal stenosis and tissue damage caused by corrosive burns when compared to the control and prednisolone-treated groups. During the initial referral of the patients with corrosive esophageal burns, for the prevention of complications as stenosis and strictures, we believe that admission of BAPN can decrease the morbidity and mortality rates. Further clinical studies are required to assess the application of BAPN in the acute treatment of corrosive burns.

## Figures and Tables

**Table 1:** Mean histopathological tissue damage score and stenosis index values.

	Mean ± SD	Min–max	Median	*P *
SI score				
Group I (control)	1.09 ± 0.26*	0.7–1.6	1.1	**0.000**
Group II (BAPN)	0.79 ± 0.19*	0.6–1.1	0.7	
Group III (prednisolone)	1.03 ± 0.38*	0.6–1.6	1.0	
Group IV (Sham)	0.44 ± 0.06	0.4–0.6	0.4	

HDS score				
Group I (control)	4.43 ± 1.13^∗#^	2.0–5.0	5.0	**0.000**
Group II (BAPN)	1.93 ± 1.02*	0.5–3.5	1.5	
Group III (prednisolone)	3.93 ± 1.02^∗#^	3.0–5.0	3.5	
Group IV (Sham)	0.00 ± 0.00	0.0–0.0	0.0	

ANOVA (Tamhane's T2 ).

*P < 0.05 difference with group IV.

^#^ *P* < 0.05 difference with group II.
